# Evaluation of safety and efficacy of an ophytrium and seboliance‐containing mousse with or without shampoo in cats with keratinisation disorders

**DOI:** 10.1111/jsap.70104

**Published:** 2026-02-26

**Authors:** C. Noli, H. Dropsy, A. Cozar, X. De Jaeger, R. Kesteman, A. Beck, R. Cristante, M. Debraine, F. Leymarios, A. Puozzo‐Barichard, M. Gatellet

**Affiliations:** ^1^ Servizi Dermatologici Veterinari Peveragno Italy; ^2^ Department of Dermatology National Veterinary School of Alfort Maisons‐Alfort France; ^3^ Ceva Santé Animale Libourne France; ^4^ Clinique Vétérinaire du Vernet Vernet France; ^5^ Clinique Vétérinaire Vetocare Bordeaux France; ^6^ Clinique Vétérinaire Feli' Santé Saint Nazaire France; ^7^ Chatdoc Clinique Bayonne France

## Abstract

**Objectives:**

This study aimed to assess the effectiveness of a protocol involving the application of topical products (DOUXO® S3 SEB Shampoo and Mousse; Ceva Santé Animale) containing ophytrium for managing feline keratinisation disorders.

**Materials and Methods:**

Nineteen client‐owned cats with a history of keratinisation disorders exhibited greasy or dry seborrhoea. Cats were treated with either a shampoo or mousse on day 0 (D0) and subsequently with the mousse every 48 to 72 hours for 3 weeks. Clinical signs were evaluated on D0, D7 and D21 using a modified Skin Seborrheic Index, which measured the percentage of body area affected, scaling, coat greasiness and secondary dermatological signs. Both veterinarians and owners provided subjective evaluations of overall skin condition and product efficacy.

**Results:**

Of the 17 cats that completed the study, SSI scores improved significantly by D7 and D21, with reductions of ≥50% in 82.4% of cats. By D21, the affected body surface had decreased by 92.9%, scaling by 70.5% and greasiness by 68.3%. Veterinarians rated the improvement as satisfactory, good or excellent in 100% of cases. The protocol was deemed effective and practical by 100% and 88.2% of owners, respectively, with 94.1% declaring an improved condition of their cats' skin and coat.

**Clinical Significance:**

The ophytrium‐containing mousse and shampoo protocol was well tolerated and effective in significantly reducing seborrhoea in cats with keratinisation disorders. High satisfaction levels were reported by both veterinarians and owners, highlighting the protocol's efficacy and practicality.

## INTRODUCTION

Skin diseases in cats are among the most prevalent reasons for seeking veterinary examination, representing 10.5% of all claims to insurance companies, with an annual incidence rate of 838 cases per 10.000 cat‐years (Hadar et al., 2023; Inoue et al., 2016). Among all dermatological conditions, keratinisation disorders represent a heterogeneous group of miscellaneous conditions affecting the epidermis, hair infundibula and sebaceous gland causing scaling, comedones, greasiness and/or malodour of the skin and coat. Underlying causes include feline acne, nutritional imbalances, congenital ichthyoses, immunomediated and paraneoplastic exfoliative reactions and sebaceous gland hyperplasia, dysplasia or inflammation. Besides feline acne, keratinisation disorders are not frequent in cats, and for this reason, there is a lack of evidence‐based publications on their management protocols.

Besides identifying and treating the underlying conditions, it is important to address the clinical signs (exfoliation and/or greasiness and/or malodour) with symptomatic therapy, to improve the skin and coat condition of the patient. This approach is particularly important in non‐curable diseases, such as congenital seborrhoeoic conditions, that need chronic symptomatic therapies. Topical products, such as shampoos, rinses, gels, creams, ointments, mousses, lotions or sprays, are particularly useful for this purpose, due to their direct action on the skin (Rosenkrantz, 2006). Protocols based on shampoos and mousses have been proven to be practical and effective both in dogs and cats (Bensignor et al., 2013; Dropsy et al., 2024; Kondratjeva et al., 2023).

There are only a few studies describing the topical treatment of feline keratinisation diseases, mainly in acne, and less frequently in other keratoseborrhoic disorders, and none of these were conducted with a product specifically formulated to treat keratinisation disorders (Bensignor, 2007; Glos et al., 2016; Sousa, 1995; Ural et al., 2008).

DOUXO® S3 SEB Shampoo and Mousse (Ceva Santé Animale, Libourne, France), containing ophytrium and seboliance, are specifically formulated for keratoseborrhoeic disorders and act by exerting kerato‐ and seboregulating actions, as recently proven in a study in dogs (Kondratjeva et al., 2023). Ophytrium is a purified natural extract derived from *Ophiopogon japonicus*, commonly known as dwarf lilyturf, mondo grass, fountainplant or monkeygrass. *In vitro* studies show that ophytrium can improve the mechanical, microbiological and immunological barriers of the skin. It enhances the amount of tight junctions, filaggrin, natural moisturising factors and ceramides, while also reducing transepidermal water loss, thus reinforcing the skin's mechanical barrier (Ollivier, Zemirline, Amalric, et al., 2019). Additionally, ophytrium inhibits the adhesion and biofilm formation of *Staphylococcus aureus* and *Staphylococcus pseudintermedius* (Ollivier, Zemirline, Marchand, et al., 2019). Seboliance is a natural product from pomegranate (Punica granatum), whose extracts have shown sebum‐regulating, anti‐exfoliative, soothing and antimicrobial properties (Barathikannan et al., 2016; BenSaad et al., 2017; Betanzos‐Cabrera et al., 2015; Lee et al., 2017; Paul et al., 2018).

The aim of this study was to evaluate the safety and efficacy of a protocol based on the application of DOUXO® S3 SEB Shampoo and Mousse (Ceva Santé Animale, Libourne, France) in the management of cats with keratinisation disorders.

## MATERIALS AND METHODS

### Study design and cat population

This was an open clinical multicentre study; animals were their own controls (pre‐ vs. post‐application), data at each follow‐up visit (D7 and D21) being compared to baseline (D0). A target number of 20 cats was considered sufficient to determine the performance and safety of the products and protocol. Considering the high variability of clinical manifestations of seborrhoea in cats and the available publication (Bensignor, 2007) where 10 cats were studied, we decided to double the number of cats.

Cats of any sex or breed were included by ECVD board‐certified or expert dermatologists (Certificate holders in dermatology) in five veterinary clinics in France, and one in Italy. The cats were client‐owned animals and stayed with their owners before, during and after the study, with constant environment, feeding (including hypoallergenic diet if applicable) and treatments, if applicable (see inclusion and exclusion criteria for concomitant therapies).

The protocol of the study was evaluated and approved by CEVA Santé Animale France Animal Ethical Committee under the CFAEC Protocol evaluation reference CFAEC‐2020‐12. The applications of the tested products are considered as “common procedures” as required by the clinical condition of the cats. The safety of the products was previously checked in acute conditions of exposure in dogs (one shampoo followed by four mousse applications per week for 3 weeks) with no problem of tolerance (Kolasa et al., 2019). The investigator informed the animal's owner about their rights and responsibilities, risks and the inconveniences regarding the study and obtained a written consent form. At any time, if the clinical status of the animals required a treatment modification, or on demand of the owner, the cats could stop the study even before the end of follow‐up as planned by the protocol.

Informed consent (verbal or written) was obtained from the owner or legal custodian of all animal(s) described in this work (experimental or non‐experimental animals, including cadavers) for all procedure(s) undertaken (prospective or retrospective studies). No animals or people are identifiable within this publication, and therefore additional informed consent for publication was not required.

### Inclusion and exclusion criteria

Included cats should have signs of keratinisation disorders, such as scaling or greasy coat, should have undergone regular ectoparasite control, with the last administered anti‐flea treatment still active and compatible with shampoo application. Dermatophytosis was excluded by Wood's lamp examination, microscopic examination of hairs and fungal hair culture (Dermatophyte Test Medium).

Exclusion criteria were: any major disease, pregnancy, lactation, presence of ectoparasitic diseases, bacterial or dermatophytic infections checked by combing, clinical examination and ancillary testing (Wood's lamp examination, microscopic examination of hairs, fungal culture, cytology). Further exclusion criteria were initiation of concurrent treatments, such as long‐lasting glucocorticoids within 8 weeks or topical steroids 4 weeks before inclusion; the use of antimicrobial or antifungal therapies, short‐acting oral glucocorticoids, antihistamines or topical antiseptics within 2 weeks before inclusion; other topical products (*e.g*. lotions, sprays, shampoos) within 1 week before inclusion. The above‐mentioned concurrent treatments were allowed if initiated before the time frames mentioned above for each drug and not modified thereafter.

### Study product and procedures

Study products were an ophytrium‐containing shampoo (DOUXO® S3 SEB shampoo, Ceva, Libourne, France) and an ophytrium‐containing mousse (DOUXO® S3 SEB mousse, Ceva, Libourne, France).

The protocol consisted of one shampoo and two mousse applications during the first week and three mousse applications per week during the two following weeks, with an interval of 48 to 72 hours between applications. The shampoo was applied to affected area(s), or to the whole body in case of generalised signs, by the veterinarian at the clinic on the day of inclusion (D0).

When the shampoo application was not possible (due to the cat's temperament or technical constraints during the D0 consultation), it was replaced by a mousse application, resulting in a mousse only application protocol during the whole study period. The mousse was applied by the owners to the affected area(s), or to the whole body in case of generalised signs, at the dose of one pump/kg for animals <6 kg and one pump/2 kg for animals more than 6 kg, according to the product specifications.

No further therapeutical intervention, neither topical nor systemic, was allowed to be initiated during the study, in particular interventions aiming at controlling the possible underlying disease causing the keratinisation disorder.

### Outcome measures

Efficacy was assessed at D0, D7 and D21 using a modified investigator‐assessed Skin Seborrhoeic Index (SSI), as described in Table 1 (Combarros et al., 2020; Noli et al., 2024; Viaud et al., 2012). At D21, veterinarians evaluated the improvement of the skin condition and the protocol efficacy from 0 (None/Absent or worse) to 5 (excellent), and questions about satisfaction with the protocol efficacy, the ease of use, the sensory characteristics of the products and the cosmetic effect on the skin and hair were asked to the owners (File S1).

**Table 1:** The modified Skin Seborrheic Index used in the study, with a score range 0 to 12

Skin Seborrheic Index	
Area affected by dermatological signs	
0	<10% of the body surface area
1	10% to 25%
2	25% to 50%
3	>50% of the body surface area
Scaling/Squamosis	
0	None
1	Mild scaling
2	High number of scales or affecting a large surface area
3	Very high
Greasy aspect of the skin/hair	
0	None
1	Mild and/or affecting a small surface area
2	Moderate and/or affecting a moderately large surface area
3	Intense and/or affecting a very large surface area
Secondary dermatological signs (alopecia, excoriations)	
0	None
1	A few signs
2	High number of signs or affecting a large surface area
3	Very high number of signs
Total score	|_|_| (sum of previous scores)

### Statistical analysis

The population used for the analysis, named as efficacy population, corresponds to the cats without major deviations. Analyses were done based on this population, except regarding safety parameters.

Demographic, efficacy, safety data and data about overall assessments were descriptively presented as percentages or mean, standard deviation, median, first and third quartiles depending on the nature of the parameter. To evaluate efficacy, comparisons on the SSI at D0 versus D7 and D0 versus D21 were performed using a Wilcoxon paired test. All secondary endpoints such as veterinarian assessment of global judgement of improvement of the skin condition, protocol efficacy and owner satisfaction assessments at the end of follow‐up were analysed descriptively.

## RESULTS

### Animal population

Nineteen cats were included in the study, two of which were excluded from the analysis due to major deviations from the protocol, leading to an efficacy population of 17 cats. One cat was lost by the owners after visit D0, and in the other, the mousse application frequency was modified due to an adverse event (diarrhoea between D7 and D21), and the application protocol was interrupted before visit at D21. In the efficacy population, 13 were European, two Birman, one Maine Coon and one Persian cat. Of these, 11 were males (64.7%, one entire) and six were females (35.3%, all neutered). Mean age was 8.3 years (range 1.4 to 17.5 years) and mean body weight was 5.5 kg (range 3.8 to 8.1 kg). Nine cats were short‐haired (of which three with thick hair) and eight were long‐haired (of which six with thick hair). Housing was indoor only for nine cats (52.9%), indoor/outdoor for seven (41.2%) and outdoor only for one cat (5.9%). Epidemiological data for all cats are provided in File S2.

No concomitant treatment was administered in any cat during the study, with the exception of those necessary to treat the adverse events described later. At D0 visit, eight cats received a shampoo (47.1%) while nine cats started the treatment protocol with a mousse application (52.9%). Shampoo was not used in these cats due to difficult temperament and/or practicality.

### Efficacy analysis

#### Evaluation of global seborrhoea

At D0, 12 cats (70.6%) had seborrhoeic signs affecting between 10% and 50% of the body surface, 8 (47.1%) showed mild or moderate greasiness of the coat and 13 (76.5%) presented scaling.

**FIG 1:**
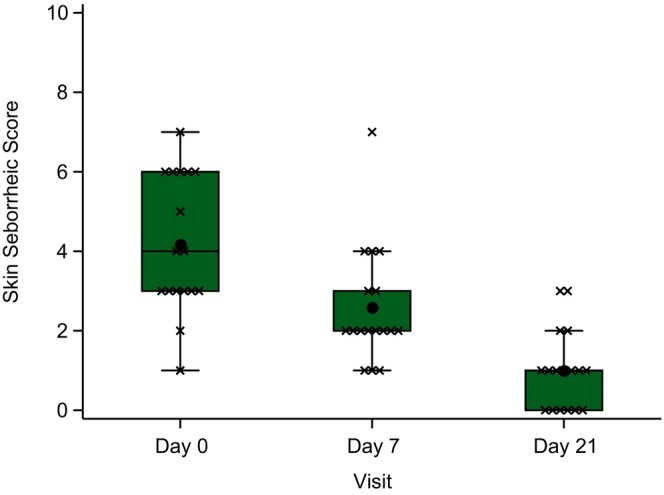
Evolution of a modified Skin Seborrheic Index in 17 cats with keratinisation disorders treated with an ophytrium‐containing shampoo ± mousse protocol. *P*‐value at D7: .001, at D21: <.001.

**FIG 2:**
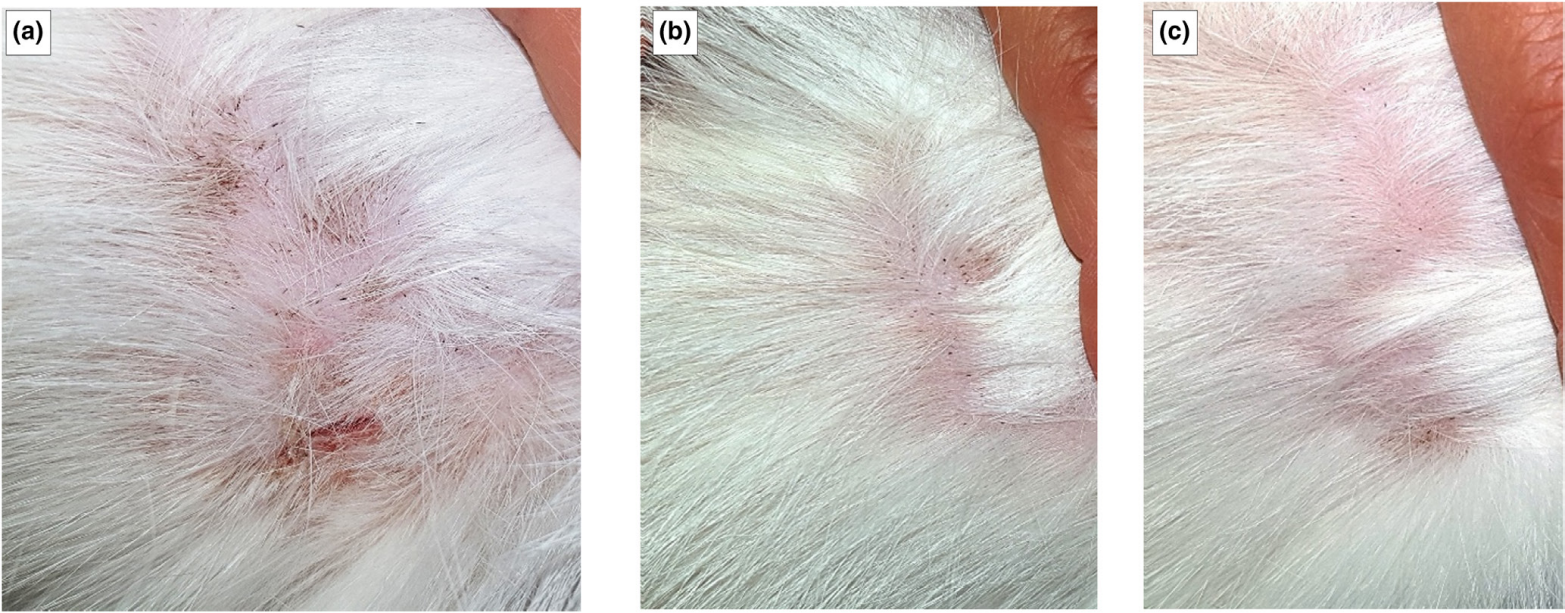
Cat affected with sebaceous adenitis: keratin plugs are evident at the basis of the hair shafts on D0 (A), while they are decreased on D7 (B) and D21 (C).

SSI median score was 4 at D0, and significantly decreased both at D7 and D21 compared to D0 (D7: *P*=.001, D21: *P*<.001), reaching a median score of 1 (range 0 to 12) after 3 weeks of mousse application. Compared to D0, at D21, the SSI score decreased by at least 50% in 14 cats (84.4%) and by at least 70% in 9 cats (52.9%) (Figs 1 and 2A–C). The individual SSI scores for each cat are provided in File S3.

#### Evolution of SSI sub‐parameters

At inclusion, more than 80% of the cats had more than 10% of their body surface area affected by keratinisation disorder signs. At Day 21, body surface area affected by keratinisation disorder signs score decreased in 82.4% of the cats, with a mean change from baseline of 92.9% (Table 2).

**Table 2:** Skin seborrhoeic sub‐score: evolution of surface area affected by dermatological signs in cats with keratinisation disorders treated with an ophytrium‐containing shampoo ± mousse protocol (*N* = 17)

Parameters	Statistics	Day 0	Day 7	Day 21
Area affected by dermatological signs				
<10% of the body surface area	% (*n*)	17.6% (3)	35.3% (6)	88.2% (15)
10% to 25%	% (*n*)	35.3% (6)	41.2% (7)	11.8% (2)
25% to 50%	% (*n*)	35.3% (6)	23.5% (4)	0.0% (0)
>50% of the body surface area	% (*n*)	11.8% (2)	0.0% (0)	0.0% (0)
Area affected by dermatological signs score compared to Day 0				
Increase (worsening)	% (*n*)		0.0% (0)	0.0% (0)
Stable	% (*n*)		58.8% (10)	17.6% (3)
Decrease (improvement)	% (*n*)		41.2% (7)	82.4% (14)

At inclusion, almost half of the cats (*n* = 8) had a high to very high number of scales. At Day 7, a majority had mild scaling, and at Day 21, two‐thirds of the cats (*n* = 11) had no scaling/exfoliation (Table 3).

**Table 3:** Skin seborrhoeic sub‐score: evolution of scaling/exfoliation in cats with keratinisation disorders treated with an ophytrium‐containing shampoo ± mousse protocol (*N* = 17)

Parameters	Statistics	Day 0	Day 7	Day 21
Scaling/squamosis				
None	% (*n*)	23.5% (4)	29.4% (5)	64.7% (11)
Mild scaling	% (*n*)	29.4% (5)	70.6% (12)	35.3% (6)
High number of scales or affecting a large surface area	% (*n*)	35.3% (6)	0.0% (0)	0.0% (0)
Very high number of scales	% (*n*)	11.8% (2)	0.0% (0)	0.0% (0)
Scaling/squamosis score compared to Day 0				
Increase (worsening)	% (*n*)		0.0% (0)	0.0% (0)
Stable	% (*n*)		47.1% (8)	35.3% (6)
Decrease (improvement)	% (*n*)		52.9% (9)	64.7% (11)

At each visit, five cats had a value of greasiness of 0. At the end of the study period, all cats had either no or only mild greasy skin/hair (maximum score given at D21 was 1) (Table 4).

**Table 4:** Skin seborrhoeic sub‐score: evolution of greasy aspect of the skin/hair in cats with keratinisation disorders treated with an ophytrium‐containing shampoo ± mousse protocol (*N* = 17)

Parameters	Statistics	Day 0	Day 7	Day 21
Greasy aspect of the skin/hair				
None	% (*n*)	41.2% (7)	41.2% (7)	64.7% (11)
Mild and/or affecting a small surface area	% (*n*)	23.5% (4)	41.2% (7)	35.3% (6)
Moderate and/or affecting a moderately large surface area	% (*n*)	23.5% (4)	11.8% (2)	0.0% (0)
Intense and/or affecting a very large surface area	% (*n*)	11.8% (2)	5.9% (1)	0.0% (0)
Greasy aspect of the skin/hair score compared to Day 0				
Increase (worsening)	% (*n*)		11.8% (2)	5.9% (1)
Stable	% (*n*)		52.9% (9)	47.1% (8)
Decrease (improvement)	% (*n*)		35.3% (6)	47.1% (8)

Thirteen cats had a “secondary dermatological signs” score of 0 at all visits, in the remaining cats secondary dermatological signs were mild at baseline and decreased at the end of the treatment (Table 5).

**Table 5:** Skin seborrhoeic sub‐score: evolution of secondary dermatological signs (excoriation, alopecia) in cats with keratinisation disorders treated with an ophytrium‐containing shampoo ± mousse protocol (*N* = 17)

Parameters	Statistics	Day 0	Day 7	Day 21
Secondary dermatological signs (excoriation, alopecia)				
None	% (*n*)	76.5% (13)	82.4% (14)	82.4% (14)
A few signs	% (*n*)	11.8% (2)	17.6% (3)	17.6% (3)
High number of signs or affecting a large surface area	% (*n*)	11.8% (2)	0.0% (0)	0.0% (0)
Very high number of signs	% (*n*)	0.0% (0)	0.0% (0)	0.0% (0)
Secondary dermatological signs (excoriation, alopecia) score compared to Day 0				
Increase (worsening)	% (*n*)		0.0% (0)	0.0% (0)
Stable	% (*n*)		82.4% (14)	82.4% (14)
Decrease (improvement)	% (*n*)		17.6% (3)	17.6% (3)

### Global assessment by veterinarians

#### Assessment of the skin condition

**FIG 3:**
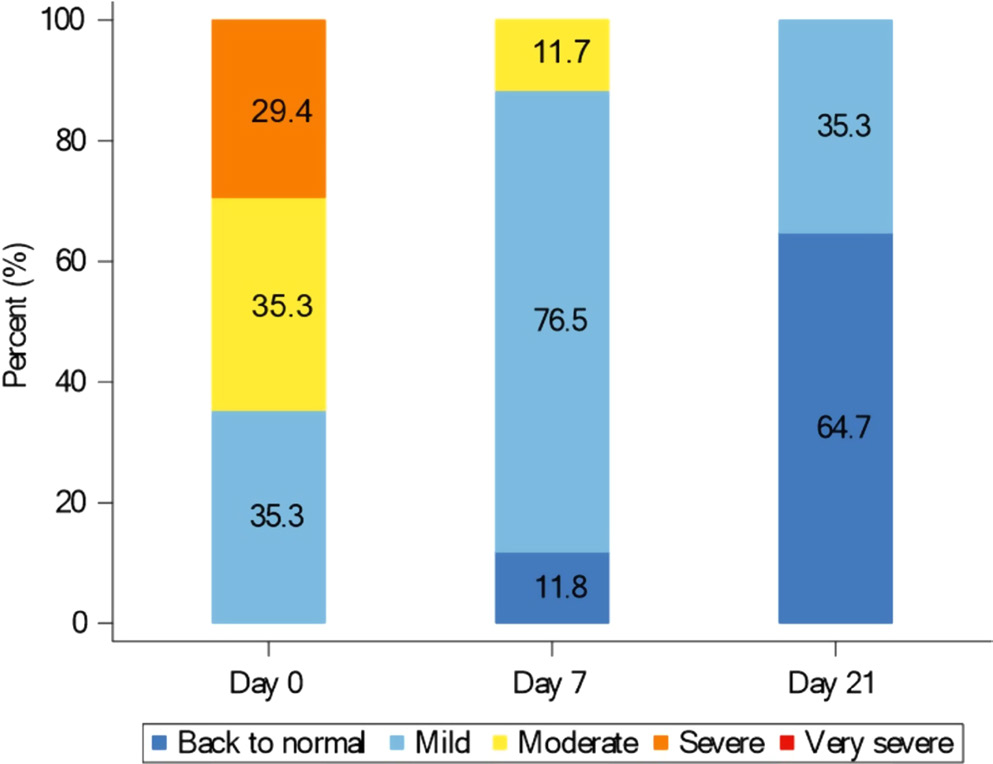
Distribution of global judgement of the severity of the skin condition in category at each visit on the PP population (*N* = 17).

At D0, one third of the cats (6/17; 35.3%) were classified as mildly affected and the same number as moderately affected. Five cats (29.4%) were judged to be severely affected (Fig 3).

At D21, 11 cats (64.7%) were considered back to normal status and 6 (35.3%) still mildly affected. From the 11 cats going back to normal at D21, 2 were severely affected at inclusion, four moderately affected and 5 mildly affected. In total, 16 of the 17 cats (94.1%) improved at D21 compared to D0 skin status (whatever their initial status) and one – mildly affected initially – remained stable over the 3 weeks; however, with fewer comedons observed on the chin, which was the only affected area.

### Efficacy of the intervention

For all cats, on a 6‐point scale (0 to 5), 100% of the veterinarians rated the efficacy between 3 and 5 (satisfactory to excellent). For six cats (35.3%), the improvement was rated as excellent. Thirteen cats (76.5%) were judged not to require any further treatment at Day 21. For the other four, the practitioners recommended:

- To continue the mousse application for a few further days due to some comedons still present (*N* = 2).
- An application of the shampoo at the groomer (*N* = 1, cat with greasy skin and tendency to scaling).
- Initiation of ciclosporin, although the improvement of cutaneous signs was qualified as good (*N* = 1). This cat presented diffuse hypotrichosis with scaling, possibly due to non‐thymoma associated feline exfoliative dermatitis.

### Global assessment by owners

**FIG 4:**
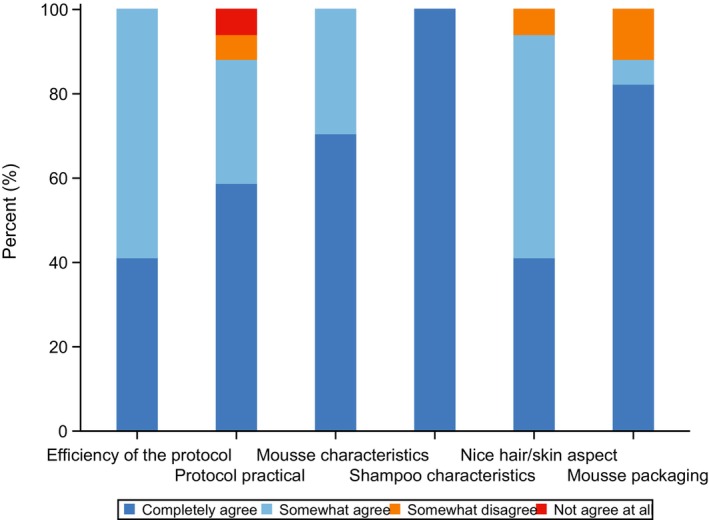
Distribution of opinion on the PP population (owner final questionnaire) (*N* = 17).

**FIG 5:**
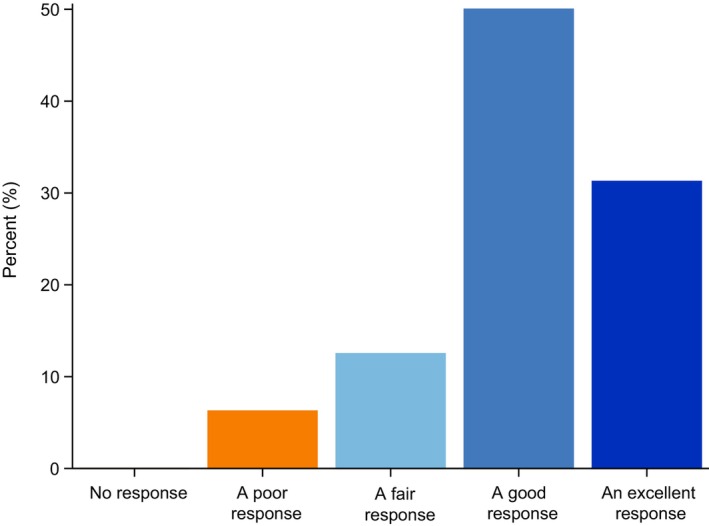
Distribution of overall response to application on the PP population (owner final questionnaire) (*N* = 16) (1 owner didn't answer this question).

All owners considered the tested protocol effective (100.0%), and almost all (88.2%) considered the protocol practical and easy to use (Fig 4). All owners liked the mousse characteristics, and the three owners whose cats received a shampoo application at D0 and answered the question about shampoo characteristics were satisfied with the product as well. All but one (94.1%) were pleased with the hair/skin aspect after product application. However, this owner was pleased with the efficacy and the ease‐of‐use of the product. Five owners (31.3%) considered the response to treatment excellent and 10 (62.5%) rated it as good or fair (Fig 5).

### Adverse events

No serious adverse event was reported in this study. Three cats presented adverse events: one cat developed two consecutive events of diarrhoea that led to interruption of the mousse applications from D8 and visit D21 was not performed. This cat was excluded from the efficacy analysis. One cat developed vomiting for 24 hours that required the administration of a concomitant treatment (maropitant 2 mg/kg once) that did not interact with the test product assessment, whose applications were continued by the owner. One further cat developed an eosinophilic dermatitis on the ventral parts of the body that was still present at the end of follow‐up. These last two cats were maintained in the efficacy analysis. Overall, topical tolerance of the mousse was good for all cats.

## DISCUSSION

This is the first study published on the use of topical products specifically developed to be applied in case of keratinisation disorders in the cat, using the same study protocol and products as already published in dogs (Kondratjeva et al., 2023).

In this study, cats were recruited based on the presence of clinical signs of keratinisation disorders, with a target sample size of 20 cats. Keratinisation disorders are not frequent in cats: they are estimated to represent only about 5% of all feline dermatological conditions (Scott et al., 2013). For this reason, it was decided to proceed with an open study, in which each animal served as its own control (pre‐ and post‐application) because the inclusion of an equally numerous placebo group was considered to be not feasible in the given study period, as well as unethical.

Keratinisation disorders can be caused by a variety of cutaneous or systemic causes. Due to the small sample size, it was decided not to stratify into subgroups depending on the underlying cause or on the specific clinical presentation, such as exfoliation, comedones, greasy hair. Instead, cats were selected based on frequently observed clinical signs that the study product aimed to improve or eliminate (scales, greasiness, *etc*.), without necessarily identifying an underlying diagnosis.

The SSI used in this study to evaluate cats with keratinisation disorders was adapted from what was proposed in prior studies conducted in dogs (Combarros et al., 2020; Noli et al., 2024; Viaud et al., 2012). No such score has ever been developed or validated for the assessment of keratinisation disorders in cats. Of the different parameters used in dogs (malodor, extension of affected area, pruritus, erythema, scaling, greasiness and secondary dermatological signs), “malodour” and “erythema” were excluded, as these are usually associated with *Malassezia* spp. dermatitis, frequent in dogs, but rare in cats (Bond et al., 2020; Crosaz et al., 2013; Hobi et al., 2024). “Pruritus” was also excluded, as it was not significant in the included cats, leaving parameters such as “extension of affected area, scaling, greasiness and secondary dermatological signs” to contribute to the assessment of the SSI used in this study.

No minimum SSI threshold was established for inclusion and no specific percentage improvement was set as the benchmark for clinical success due to the absence of a validated scoring system and of scientific consensus. Instead, a statistically significant improvement of the score was the target defined in this study. At baseline, the SSI score was moderate (mean of 4.2 on a maximum score of 12). Despite this moderately low value at D0, the tested protocol significantly improved the skin condition at D7 and D21. In our study, the combined SSI score as well as all single sub‐parameters decreased by D21 by much more than 50%, defined as “intervention success” by some investigators in other studies (Bensignor, 2007). The author analysed in a 0 to 4 scoring system the amount of scales, the dryness or greasiness of the skin and the coat quality, obtaining satisfactory results in 90% of cats within 4 weeks (Bensignor, 2007). Even if products and frequency of application in this and in our study were different, one can conclude that in general topical application can be a useful, effective and practical intervention to improve coat and skin quality in cats with keratoseborrhoeic disorders. A recent study in dogs, using the same protocol and products as our study, albeit with a slightly different SSI scoring system and performing a shampoo in every animal, also showed a significant SSI reduction, suggesting that this protocol may be useful in either species (Kondratjeva et al., 2023).

Cat‐friendly veterinary medicine should try to avoid sources of stress as much as possible during the veterinary consultation and treatment (Rodan et al., 2022). A veterinary visit can be very stressful for cats. If cats were too stressed to further receive a shampoo at the clinic on D0, the shampoo was replaced by mousse. As a matter of fact, about half (8/17) of the animals included in our study received a shampoo on D0 and 9/17 received the mousse; this difference in application could theoretically have influenced the results. A direct comparison between the two groups was not performed due to the low number of cases and consequent low statistical power; instead, it was chosen to add the first application (shampoo or mousse) as a variable in a model on the absolute SSI change. This variable did not affect the results, so that all cats were analysed together. The same approach was recently applied in a similar study on the performance of applications of ophytrium‐containing mousse with or without shampoo in cats with pruritic and irritated skin (Dropsy et al., 2024).

Veterinarians performed a subjective global assessment of the cats' skin condition, not directly linked to SSI, categorising it as normal, mildly affected, moderately affected, severely affected or very severely affected at each visit. In line with what already observed with a similar protocol in allergic cats, by D21 veterinarians declared an improvement of skin condition in 94.1% of cats (16/17), regardless of the initial severity (Dropsy et al., 2024). Furthermore, 100% of veterinarians rated the efficacy between 3 and 5 (satisfactory to excellent), indicating that topical applications could be an effective tool for the management of feline keratinisation disorders. However, as some of the cats (4/17) required further interventions at the end of the study, it needs to be reminded that the current protocol alone may not suffice in all cases but could represent a useful adjunct treatment strategy.

The majority (88.2%) of owners found the mousse easy to apply and the protocol practical. Being able to avoid shampooing and replacing it with a mousse application may be very helpful to avoid stressful experiences in cats. One owner complained about the greasy/sticky appearance of the coat after the mousse application, possibly because the hair was not brushed thereafter. It is thus important to remind owners to brush the coat after application, as recommended by the manufacturer, in order to avoid subsequent excessive licking and the sticky appearance that could bother the owner.

The products used in this study demonstrated excellent local tolerance, no systemic and no buccal toxicity from self‐licking, as already confirmed by previous studies in cats and in dogs (Dropsy et al., 2024; Kolasa et al., 2019). The few adverse events observed in the current study were not considered to be related to the product and did not, with the exception of one case, lead to protocol interruption.

In conclusion, this study supports the safety and performance of a protocol combining one application of DOUXO® S3 Seb shampoo followed by DOUXO® S3 Seb mousse applications every 2 to 3 days for 3 weeks in the management of cats with keratinisation disorders. The alternative protocol, which replaces the initial shampoo with a first application of DOUXO® S3 Seb Mousse, was equally effective, so that study results seem to support both approaches.

## Author contributions

**H. Dropsy:** Investigation. **A. Cozar:** Conceptualization; methodology; validation; project administration. **C. Noli:** Investigation; writing – original draft; writing – review and editing. **Xavier De Jaeger:** Conceptualization; methodology; validation; project administration. **R. Kesteman:** Data curation; formal analysis. **A. Beck:** Data curation; formal analysis. **R. Cristante:** Investigation. **M. Debraine:** Investigation. **F. Leymarios:** Investigation. **A. Puozzo‐Barichard:** Investigation. **M. Gatellet:** Conceptualization; methodology; validation; project administration.

## Conflict of interest

A. Cozar, M. Gatellet, R. Kesteman, A. Beck, X. De Jaeger are employees of Ceva Santé Animale, Libourne, France. The remaining authors received financial support from Ceva Santé Animale for the research, authorship and publication of this article.

## Funding

This study was supported by Ceva Santé Animale, Libourne, France.

## Supporting information


File S1.

File S2.

File S3.

## Data Availability

The data that support the findings of this study are available on request from the corresponding author. The data are not publicly available due to privacy or ethical restrictions.
