# Supplementary material for: Evaluation of safety and efficacy of an ophytrium and seboliance‐containing mousse with or without shampoo in cats with keratinisation disorders
Source: J Small Anim Pract. 2026 Feb 26;67(7):619–26. doi: 10.1111/jsap.70104 (PMC13327231; doi:10.1111/jsap.70104)
Supplement: Supplementary file 1 — File S1. [file JSAP-67-619-s002.docx]

OWNER FINAL QUESTIONNAIRE

- The protocol (shampoo-mousse or just mousse) 3 times weekly is effective:
  - I totally disagree
  - I partially disagree
  - I partially agree
  - I totally agree
- The protocol (shampoo-mousse or just mousse) 3 times weekly is practical and easy to implement:
  - I totally disagree
  - I partially disagree
  - I partially agree
  - I totally agree
- The haircoat and skin of my cat have a nice aspect (healthy, soft and shiny)
  - I totally disagree
  - I partially disagree
  - I partially agree
  - I totally agree
- How would you evaluate the global response to the products?
  - No response
  - Mild response
  - Good response
  - Excellent response
- I love the products’ characteristics

| Mousse | Shampooing |
| --- | --- |
| - - I totally disagree   - I partially disagree   - I partially agree   - I totally agree | - - I totally disagree   - I partially disagree   - I partially agree   - I totally agree |

- The mousse’s packaging (design and pump) is very practical and make the application easy:
  - I totally disagree
  - I partially disagree
  - I partially agree
  - I totally agree
- Regarding the protocol (shampoo-mousse or just mousse) 3 times weekly applid to my cat, I am globally:
  - Not satisfied
  - Not fully satisfied
  - Satisfied
- Very satisfied

Any comments regarding efficacy, cosmetic and practical aspects of the protocol:
