# Supplementary material for: Evaluation of safety and efficacy of an ophytrium and seboliance‐containing mousse with or without shampoo in cats with keratinisation disorders
Source: J Small Anim Pract. 2026 Feb 26;67(7):619–26. doi: 10.1111/jsap.70104 (PMC13327231; doi:10.1111/jsap.70104)
Supplement: Supplementary file 2 — File S2. [file JSAP-67-619-s001.docx]

**Supplementary file 2**: Epidemiological data of the included cats.

| **Animal #** | **Breed** | **Sex** | **Age (years)** | **Body weight (kg)** | **Coat length** | **Coat dense** | **Housing** |
| --- | --- | --- | --- | --- | --- | --- | --- |
| 1 | Crossbreed | FN | 7.0 | 8.1 | Long | Yes | Indoor only |
| 2 | Crossbreed | FN | 8.1 | 4.8 | Short | Yes | Indoor and outdoor |
| 3 | Birman | MC | 9.1 | 5.2 | Long | No | Indoor and outdoor |
| 4 | Crossbreed | MC | 4.0 | 6.4 | Short | No | Indoor and outdoor |
| 5 | Persan | MC | 3.2 | 4.5 | Long | Yes | Indoor only |
| 6 | Crossbreed | MC | 4.2 | 6.2 | Short | Yes | Indoor and outdoor |
| 7 | Crossbreed | MC | 9.4 | 5 | Short | No | Outdoor only |
| 8 | Crossbreed | FN | 5.1 | 4.6 | Long | No | Indoor and outdoor |
| 9 | Crossbreed | MC | 8.5 | 6.6 | Short | No | Indoor only |
| 10 | Maine coon | MC | 11.6 | 7 | Long | Yes | Indoor only |
| 11 | European | FN | 11.9 | 7.7 | Short | No | Indoor only |
| 12 | Crossbreed | MC | 9.6 | 4.5 | Long | Yes | Indoor only |
| 13 | Birman | MI | 1.4 | 3.8 | Long | Yes | Indoor only |
| 14 | European | MC | 4.0 | 5.8 | Short | No | Indoor only |
| 15 | European | MC | 17.5 | 4 | Short | No | Indoor only |
| 16 | Crossbreed | FN | 12.3 | 5.5 | Long | Yes | Indoor and outdoor |
| 17 | Crossbreed | FN | 14.1 | 4 | Short | Yes | Indoor and outdoor |

FN: female neutered; MC: male castrated
