# Supplementary material for: Evaluation of safety and efficacy of an ophytrium and seboliance‐containing mousse with or without shampoo in cats with keratinisation disorders
Source: J Small Anim Pract. 2026 Feb 26;67(7):619–26. doi: 10.1111/jsap.70104 (PMC13327231; doi:10.1111/jsap.70104)
Supplement: Supplementary file 3 — File S3. [file JSAP-67-619-s003.docx]

| **Supplementary file 3**: clinical scores of the included cats | | | | | | | | | | | | | | | |
| --- | --- | --- | --- | --- | --- | --- | --- | --- | --- | --- | --- | --- | --- | --- | --- |
| **Animal #** | **Area affected by dermatological signs** | | | **Scaling** | | | **Greasy aspect of the skin /hair** | | | **Secondary dermatological signs (excoriation, alopecia)** | | | **Total Skin Seborrheic Index** | | |
|  | **D0** | **D7** | **D21** | **D0** | **D7** | **D21** | **D0** | **D7** | **D21** | **D0** | **D7** | **D21** | **D0** | **D7** | **D21** |
| 1 | 3 | 2 | 0 | 2 | 1 | 1 | 1 | 1 | 1 | 0 | 0 | 0 | 6 | 4 | 2 |
| 2 | 2 | 0 | 0 | 1 | 1 | 0 | 0 | 0 | 0 | 0 | 0 | 0 | 3 | 1 | 0 |
| 3 | 2 | 1 | 0 | 1 | 0 | 0 | 3 | 1 | 1 | 0 | 0 | 0 | 6 | 2 | 1 |
| 4 | 1 | 1 | 0 | 1 | 1 | 0 | 0 | 0 | 0 | 0 | 0 | 0 | 2 | 2 | 0 |
| 5 | 1 | 0 | 0 | 1 | 1 | 1 | 1 | 1 | 0 | 0 | 0 | 0 | 3 | 2 | 1 |
| 6 | 1 | 1 | 0 | 1 | 1 | 1 | 1 | 1 | 0 | 0 | 0 | 0 | 3 | 3 | 1 |
| 7 | 2 | 2 | 1 | 2 | 1 | 1 | 0 | 3 | 0 | 2 | 1 | 1 | 6 | 7 | 3 |
| 8 | 1 | 1 | 0 | 0 | 0 | 0 | 2 | 0 | 0 | 2 | 1 | 1 | 5 | 2 | 1 |
| 9 | 1 | 1 | 0 | 2 | 1 | 0 | 0 | 0 | 0 | 1 | 0 | 0 | 4 | 2 | 0 |
| 10 | 1 | 0 | 0 | 2 | 1 | 0 | 0 | 0 | 0 | 0 | 0 | 0 | 3 | 1 | 0 |
| 11 | 2 | 2 | 0 | 2 | 1 | 0 | 2 | 0 | 0 | 0 | 0 | 0 | 6 | 3 | 0 |
| 12 | 2 | 2 | 0 | 3 | 1 | 1 | 2 | 1 | 0 | 0 | 0 | 0 | 7 | 4 | 1 |
| 13 | 0 | 0 | 0 | 0 | 0 | 0 | 3 | 2 | 1 | 0 | 0 | 0 | 3 | 2 | 1 |
| 14 | 3 | 1 | 0 | 3 | 1 | 0 | 0 | 0 | 0 | 0 | 0 | 0 | 6 | 2 | 0 |
| 15 | 2 | 1 | 1 | 2 | 1 | 1 | 0 | 2 | 1 | 0 | 0 | 0 | 4 | 4 | 3 |
| 16 | 0 | 0 | 0 | 0 | 0 | 0 | 2 | 1 | 1 | 1 | 1 | 1 | 3 | 2 | 2 |
| 17 | 0 | 0 | 0 | 0 | 0 | 0 | 1 | 1 | 1 | 0 | 0 | 0 | 1 | 1 | 1 |
